# Matrix metalloproteinase-2 (MMP-2) is associated with survival in breast carcinoma

**DOI:** 10.1038/sj.bjc.6601238

**Published:** 2003-09-30

**Authors:** A Talvensaari-Mattila, P Pääkkö, T Turpeenniemi-Hujanen

**Affiliations:** 1 Department of Obstetrics and Gynecology, PO Box 5000, 90014, University of Oulu, Oulu, Finland; 2 Department of Pathology, PO Box 5000, 90014, University of Oulu, Oulu, Finland; 3 Department of Oncology and Radiotherapy, PO Box 5000, 90014, University of Oulu, Oulu, Finland

**Keywords:** gelatinase A, 72 kDa type IV collagenase, metastasis, invasion

## Abstract

Adjuvant therapy is one of the major advances in the treatment of breast carcinoma patients – but do all patients need it? New predictive markers, which are able to save breast carcinoma patients from the most toxic adjuvant therapies, are still needed. The expression of matrix metalloproteinases (MMP-2) has been previously linked to invasiveness of carcinoma cells. In this study, we explored the role of MMP-2 as a prognostic factor in breast carcinoma in a large series to be able to show the favourable effect of MMP-2 negativity in poor prognosis subgroup of hormone receptor-negative patients. The MMP-2 immunoreactive protein was evaluated from primary adenocarcinoma of the breast in 453 cases by using a specific monoclonal antibody in immunohistochemical stainings. The MMP-2 protein found in breast carcinoma tumour cells was here shown to be associated with a shortened recurrence-free survival or relative overall survival (*P*=0.03). It was shown here that MMP-2 negativity is significantly linked to favourable prognosis in patients considered to be at risk due to their hormone receptor negativity. In the patient group presenting with a progesterone receptor-negative tumour, the survival rate of the MMP-2-positive cases was 58% while it was 95% in MMP-2-negative cases after 10 years of follow-up (*P*=0.005). The present data shows for the first time that MMP-2 negativity could serve as a marker for favourable prognosis in breast carcinoma patients with a hormone receptor-negative tumour usually associated with high risk. MMP-2 is also shown to correlate to shortened survival independent of major prognostic indicators in patients with primary breast carcinoma.

Breast carcinoma is the most frequent malignancy among women in Finland as well as in other Western countries (Finnish Cancer Registry, 1995; Parkin *et al*, 1997). It is a pathologically and clinically heterogenous disease with variable prognosis. Breast carcinomas are potentially highly malignant tumours due to their capacity to invade locally and to metastasise. The traditional prognostic factors of breast carcinoma include the size of the primary tumour, axillary lymph node involvement, tumour grade, oestrogen or progesterone receptor status of the primary tumour and menopausal status of the patients.

Tumour invasion and metastasis are the major causes of treatment failure or death for carcinoma patients. The role of matrix metalloproteinases (MMPs) in tumour invasion and metastasis as well as in tumour angiogenesis is important. Matrix metalloproteinase-2 (MMP-2/gelatinase A/72-kDa type IV collagenase) is a member of zinc-dependent endopeptidases that degrade matrix proteins, among other type IV collagens in basement membranes (Liotta and Stetler-Stevenson, 1990; Liotta *et al*, 1998; Curran and Murray, 1999). The expression of MMP-2 has been strongly associated with the progression of malignancy of several types of carcinoma (D'Errico *et al*, 1991; Levy *et al*, 1991; Ellenrieder *et al*, 2000; Sakata *et al*, 2000; Giannelli *et al*, 2002). In primary skin melanoma (Väisänen *et al*, 1998), lung carcinoma (Kodate *et al*, 1997), ovarian carcinoma (Davidson *et al*, 1999) and brain neoplasms (Jäälinoja *et al*, 2000), the expression of the immunoreactive protein for MMP-2 was associated with a poor prognosis. In several studies, MMP-2 has been shown to be expressed in breast carcinoma (Liotta and Stetler-Stevenson, 1990; Monteagudo *et al*, 1990; D'Errico *et al*, 1991; Davies *et al*, 1993; Tryggvason *et al*, 1993; Iwata *et al*, 1996; Garbett *et al*, 1999, 2000; Jones *et al*, 1999) and it has been localised in breast carcinoma cells using immunohistochemical methods (Daidone *et al*, 1991; Höyhtyä *et al*, 1994). In limited series, MMP-2 positivity is associated with unfavourable prognosis in both premenopausal and postmenopausal node-positive breast carcinoma patients (Talvensaari-Mattila *et al*, 1998, 1999, 2001).

This study is aimed at defining the possible favourable effect of the MMP-2 negativity in primary breast carcinoma in high-risk patient groups while confirming the MMP-2 immunoreactive protein as a prognostic factor also in node-negative breast carcinoma.

## MATERIALS AND METHODS

Breast tissue samples were from the primary tumours of 453 patients operated on during the years 1981–1995 in Northern Finland. The formalin-fixed, paraffin-embedded blocks were obtained from the files of the Departments of Pathology, Oulu University Hospital and the Central Hospitals of Kajaani, Kemi, Kokkola and Rovaniemi. The minimum follow-up time was 60 months (range 60–150 months).

Stage, tumour size and axillary node involvement of breast carcinoma were determined according to the UICC TNM classification (Hermanek and Sobin, 1992). The tumours were classified according to the World Health Organization's International Classification of Breast Tumors (Scarff and Torloni, 1986). The ductal carcinomas were graded (I–III) by evaluating tubule formation, nuclear pleomorphism and the mitotic rate according to the criteria of Bloom and Richardson (1957).

The patients were 26–85 years of age, the median age being 52 years. Ductal infiltrating carcinoma was the most frequent histological type. In this material, the number of the node-positive cases is over-represented, 302 out of 453 patients, to increase the power to test the effect of MMP-2 negativity in advanced breast carcinoma. A small tumour sample taken during the operation was used for routine steroid receptor assays. Both oestrogen and progesterone receptor charcoal assays were performed in 334 cases. In all, 65 tumours were both oestrogen and progesterone receptor negative. Mastectomy with axillary evacuation was the primary treatment in most of the cases, one case remained inoperable.

All patients without distant metastases and with histologically positive axillary lymph nodes, regardless of the number of nodes or size of the primary tumour, received postoperative radiotherapy covering the axillary, supraclavicular and internal mammary lymph nodes and the chest wall around the mastectomy scar. Adjuvant antioestrogen therapy had been used in 138 cases, most of them with a stage II or III disease, and adjuvant cyclophosphamide–methotrexate–fluorouracil (CMF) chemotherapy in 104 cases or FEC (5-fluorouracil-epirubicin-cyclophosphamide) in 96 cases. Patients with metastatic disease (M1) were operated (except one) and local radiotherapy was given. Additionally, the patients received antioestrogen therapy (10 patients) or chemotherapy (two patients). Recurrences in patients with receptor-positive tumour were treated primarily with hormonal therapy.

### Immunohistochemical staining

The histologic material fixed in 10% formalin and embedded in paraffin was cut into 4 *μ*m slides and they were incubated for 12 h at 37°C, dewaxed in a histological clearing agent, Histo-Clear (National Diagnostics, Atlanta, GA, USA), and hydrated. The specimens were treated with 0.4% pepsin (Sigma, St Louis, MO, USA) for 20 min at 37°C. The avidin–biotin–immunoperoxidase technique was used according to Hsu *et al*, 1981. Mouse monoclonal antibody (CA-4001; Diabor Ltd, Oulu, Finland) against MMP-2 was used as a primary antibody. The antibody has been previously shown to detect the latent (inactive), 70–72 kDa form of MMP-2. The specificity has been confirmed by a Western blot analysis (Höyhtyä *et al*, 1994). Endogenous peroxidase activity was blocked by incubating the slides in 3% hydrogen peroxide in absolute methanol for 15 min, and nonspecific binding was blocked with 10% goat serum for 15 min.

The specimens were incubated for 60 min at room temperature in a humidity chamber, and immunohistological staining was continued using a Histostain-bulk kit (Zymed, San Francisco, CA, USA) according to the manufacturer's instructions. Biotinylated anti-mouse IgG served as a second antibody, and the peroxidase was introduced using a streptavidin conjugate. The slides were washed thoroughly with phosphate-buffered saline between each stage in the procedure. The antibody reaction was visualised using a fresh substrate solution containing an aminoethyl carbazole substrate kit (AEC, Sigma). The sections were counterstained with haematoxylin, dehydrated and mounted in glycerol–vinyl–alcohol (GVA mount, Zymed). For the negative controls, the primary antibody was replaced with mouse nonimmuno IgG. For the positive controls, we used previously known MMP-2-positive specimens of breast carcinoma.

### Evaluation of the MMP-2 immunostaining

A section was considered negative or positive according to the absence or presence of positive staining of the neoplastic cells. The staining was scored as follows: no positive cells, less than 50% of the neoplastic cells staining positive (MMP-2+) and >50% of the neoplastic cells positive (MMP-2++).

Immunostaining for MMP-2 was scored by three independent observers. Only cases giving repeatable scores in immunostaining were included in the data. The clinical data were collected and analysed after the evaluation of the immunostaining scores for a given case. The scoring of the immunoreaction and collecting the clinical data were performed independently without knowledge of each other.

### Statistical analysis

The score for MMP-2 immunoreactivity was compared with other prognostic variables by the *χ*^2^ method. *P*-values <0.05 were considered statistically significant.

Survival was defined as the time elapsing from the primary operation to the date of death due to breast carcinoma. The recurrence-free survival (RFS) was determined as time in months from the date of diagnosis to the date of local recurrence or metastasis.

Survival rates were analysed by the Kaplan–Meier method (Kaplan and Meier, 1958) for up to 10 years of the follow-up. Differences between the subgroups were compared by means of a log-rank test (Mantel, 1966) for up to 10 years. The effect of MMP-2 positivity on survival was analysed in various subgroups representing the major prognostic variables recognised in breast carcinoma.

The multivariate analysis was tested with the Cox's regression model of survival time (Cox, 1972). BMDP statistical software (University of California Press, Berkeley, CA, USA) was used (Dixon *et al*, 1990).

## RESULTS

**Figure 1:**
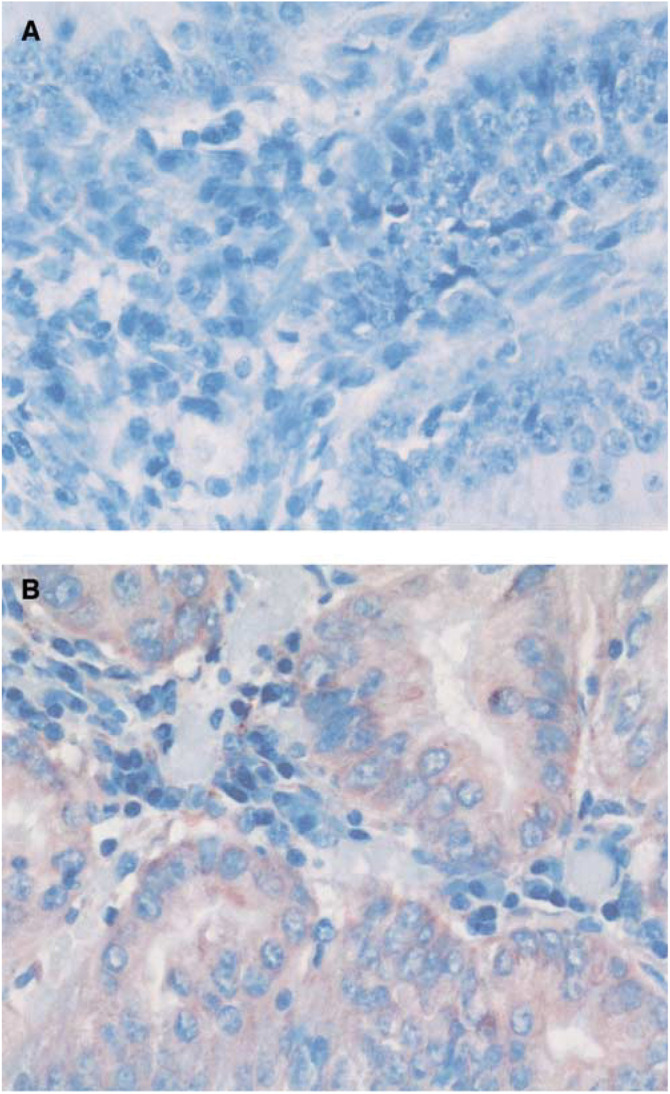
Cytoplasmic immunostaining of MMP-2 in primary breast carcinoma. Immunostaining was performed as described in Materials and Methods by using an anti-MMP-2 monoclonal antibody: (**A**) negative (−), (**B**) positive (++) case.

In this study, intracytoplasmic staining of the protein for MMP-2 was found in 78% of the primary tumours of breast carcinoma, 50% displaying an extensive positivity (>50% of the tumour cells positive; MMP-2++). Negative staining (MMP-2) was found in 22% of the primary tumours of breast carcinoma. The immunoreactive protein in carcinoma cells localised to the cytoplasm (Figure 1A, B

). There was no correlation between the MMP-2 protein expression and stage, grade or hormone receptor status (Table 1

**Table 1:** Expression of MMP-2 immunoreactive protein in breast carcinoma

		**MMP-2 positive**	
	***n***	***n***	
*All patients*	453	354	(78%)
Age (years)	26–85		
T1–2	404	318	(79%)
T3–4	49	36	(73%)
N0	152	123	(81%)
N1–2	301	231	(77%)
M0	439	341	(78%)
M1	14	13	(93%)
			
Histology			
*Ductal infiltrating*			
Grade I	39	28	(72%)
Grade II–III	302	240	(80%)
*Oestrogen receptor*			
Positive	262	199	(76%)
Negative	72	61	(85%)
Unknown	119		
*Progesterone receptor*			
Positive	236	181	(77%)
Negative	99	79	(80%)
Unknown	118		
*Adjuvant treatment*			
No	187	154	(82%)
Yes	226	200	(88%)

).

**Figure 2:**
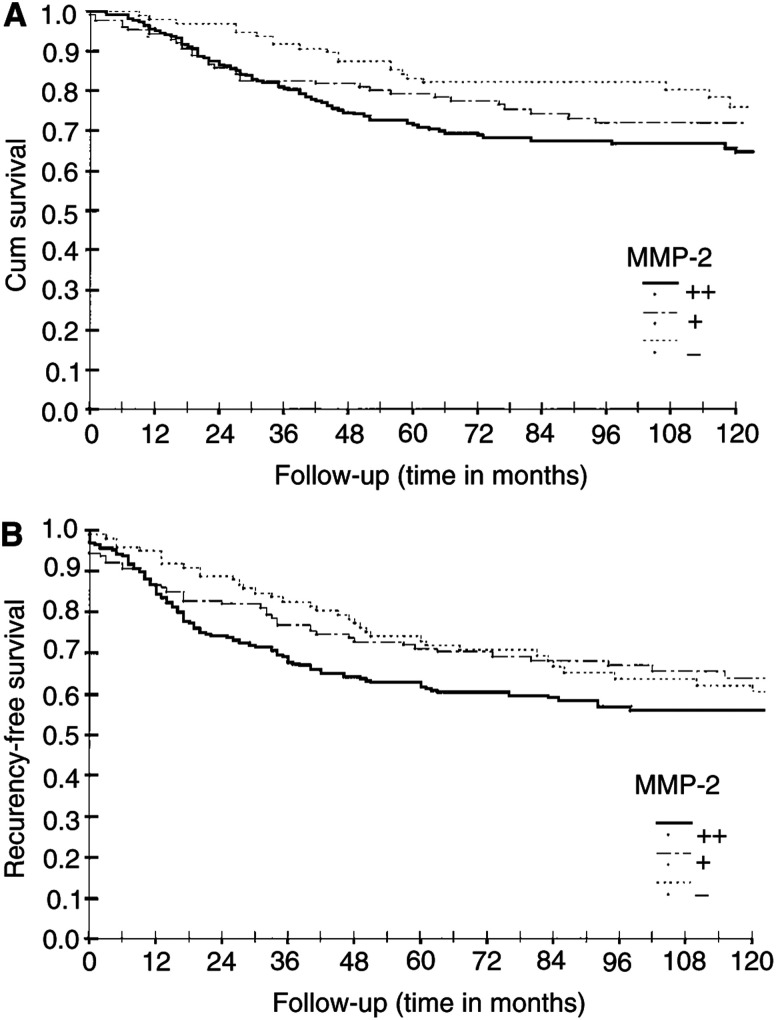
Survival analysis (Kaplan–Meier) of the breast carcinoma patients according to the MMP-2 immunoreactivity of the primary tumour: (**A**) overall survival, (**B**) RFS. MMP-2 negative (−), MMP-2 positivity; weak (+), strong (++). Log-rank analysis of MMP-2 negative *vs* strong positive: (**A**) *P*=0.03, (**B**) *P*=0.05.

A statistically significant correlation between MMP-2 positivity and overall survival was found in this study. The 10-year overall survival was 72% in patients with an MMP-2 immunoreactive protein positive (+) and 64% in patients with an MMP-2 immunoreactive protein strongly positive (++) breast carcinoma, compared to 77% in the patient group with an MMP-2-negative primary tumour, the 10-year RFS being 60, 56 and 64%, respectively (Figure 2A, B

).

In the node-negative patient group, all patients with MMP-2-negative immunostaining were alive in the follow-up time of 10 years. In the patient group with a positive immunohistochemical staining for MMP-2, the overall survival was 87% (*P*=0.03, Table 2

**Table 2:** Recurrence-free and overall survival of breast carcinoma patients according to MMP-2 staining in different patient groups

	**MMP-2 immunoreaction**			
	**Recurrence-free survival**		**Overall survival**	
	**−**	**+/++**	**−**	**+/++**
All patients	64	59	77	68***
T1–2	66	60	80	70**
				
Node negative	79	78	100	87****
Node positive	58	48*****	61	56*****
				
*Oestrogen receptor*				
Negative	90	56*****	90	58*****
Positive	55	55	75	67**
*Progesterone receptor*				
Negative	80	56**	95	58*
Positive	55	55	71	67
Grade II–III	57	48*****	64	59****

* *P*=0.005,

** *P*=0.02,

*** *P*=0.03,

**** *P*=0.04,

***** *P*<0.05. The surviving fraction is given as % at 10 years in Kaplan–Meier analysis.

).

**Figure 3:**
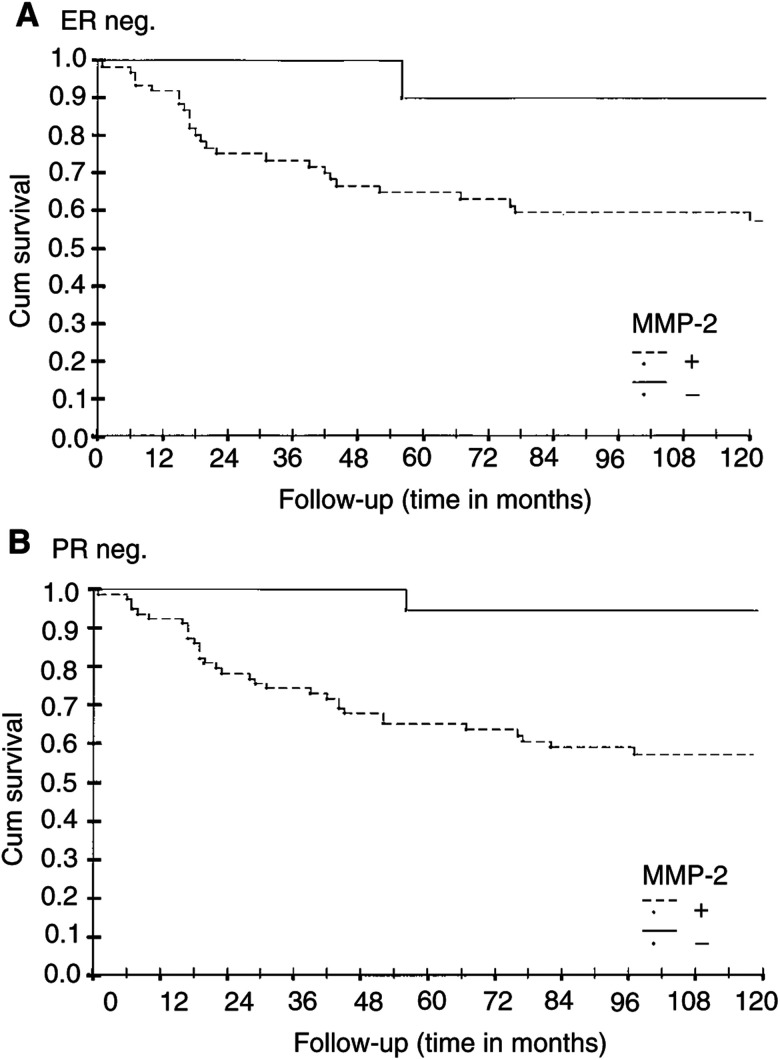
Relative survival of the breast carcinoma patients according to the MMP-2 immunoreactivity of the primary tumour in hormone receptor-negative patient groups: (**A**) oestrogen receptor negative, (**B**) progesterone receptor negative patient groups. Log-rank analysis of MMP-2 negative *vs* positive. (**A**) *P*=0.05, (**B**) *P*=0.005.

Out of 334 breast carcinoma patients, 72 were negative for oestrogen receptors and 96 for progesterone receptors. In these patients, the MMP-2 positivity indicated a very unfavourable prognosis. In all, 58% of the patients presenting with MMP-2-positive and oestrogen (*n*=59) or progesterone (*n*=96) receptor-negative primary tumour were alive after the 10 years of follow-up. In contrast, 90% of the patients with an MMP-2-negative, oestrogen receptor-negative (*n*=12) tumour and 95% of those with an MMP-2 negative, progesterone receptor-negative (*n*=20) tumour were alive at that time (Figure 3A, B

). The Kaplan–Meier analysis showed that the RFS was 90 or 80% in patients with an MMP-2-negative, oestrogen receptor-negative or progesterone receptor-negative primary tumour, respectively, while it was 56% in the MMP-2-positive tumours in both oestrogen- or progesterone receptor groups (*P*=0.047, 0.023).

In grade 2 and 3, tumours MMP-2 positivity correlated significantly with shortened overall survival (*P*=0.04). The 10-year RFS of the patients with MMP-2-negative primary tumour was 57%, whereas it was 48% in those patients with a tumour displaying MMP-2 positivity (*P*=0.05), the overall survival being 64% *vs* 59%, respectively.

A multivariate analysis was conducted to evaluate further whether the correlation between MMP-2 positivity and shortened survival could be related to the association of MMP-2 with other prognostic factors. The results showed that MMP-2 positivity in carcinoma cells maintained its association with a poor outcome (Table 3

**Table 3:** Cox's regression model of survival time

	***n*=452**	***β***	**s.e.**	***P***	**OR**	**95% Cl for *β***	**OR's *P*-value**
*MMP-2*				0.02			
Negative	99				1.00		
Positive	353	0.579	0.245		1.78	1.10–2.88	0.02
							
*Age* (years)				0.02			
<55	266				1.00		
>55	186	0.445	0.183		1.56	1.09–2.23	0.02
							
*Tumour*				0.001			
1–2	403				1.00		
3–4	49	1.106	0.223		3.02	1.95–4.67	0.001
							
*Nodes*				0.001			
0	152				1.00		
1–2	300	1.375	0.285		3.95	2.26–6.91	0.001
							
*Grade*				0.01			
I	39				1.00		
II–III	302	1.923	0.715		6.84	1.68–27.8	0.01

*β*=coefficient; s.e.=standard error of the mean; OR=odds ratio.

). MMP-2 positivity appeared to increase the risk of death 1.8-fold during the first 10 years of follow-up.

## DISCUSSION

The expression of MMP-2 immunoreactive protein has been associated with invasive and metastatic tumours in previous *in vitro* studies (Liotta *et al*, 1980; Garbisa *et al*, 1987; Nakajima *et al*, 1987; Bernhard *et al*, 1990). In this study, intracytoplasmic expression of the protein for MMP-2 was found in 78% of the primary tumours of breast carcinoma. The amount of positive cases is in line with previously published data (Monteagudo *et al*, 1990; Daidone *et al*, 1991; Visscher *et al*, 1994; Talvensaari-Mattila *et al*, 1998, 1999, 2001; Lee *et al*, 1996). This study constitutes the largest material of breast carcinoma published showing the prognostic value of MMP-2.

In this material consisting of 453 cases, the MMP-2 immunoreactive protein was able to predict a relapse during the 10 years of the follow-up. The 10-year RFS was 60% in patients with a low-grade (+) and 56% in those with a strongly positive (++) tumour, compared to 64% in patients with an MMP-2-negative tumour. Also, the 10-year overall survival rate was significantly inferior among these patients. These differences are high enough to be also clinically significant, and especially the differences between − and ++ groups suggest that the MMP-2 immunoreactive protein is worthy of careful evaluation as a possible marker for biologic aggressiveness in breast carcinoma patients. Further, the previous studies have failed to show any statistically significant differences in survival between the patients with MMP-2-negative *vs* -positive primary tumours in node-negative patient group. Here the patient group presenting an MMP-2-negative primary breast carcinoma without a lymph node involvement enjoyed an excellent prognosis for survival, 100% of the patients being alive after 10 years of follow-up (Table 2). No difference was, however, found in the RFS suggesting that MMP-2 negativity may be associated with better responses to treatment of the metastatic disease and/or show progression of the disease.

The patients with an MMP-2-negative breast carcinoma form a relatively large patient group when the incidence of breast carcinoma is taken into consideration. It is possible that the lack of MMP-2 could become an important factor in certain subgroups of breast carcinoma when selecting the adjuvant therapy. A well-known risk factor in breast carcinoma is hormone receptor negativity. It is interesting that 90% of the patients with an oestrogen receptor negative, MMP-2 negative or 95% of the patients with a progesterone receptor negative, MMP-2 negative primary tumour were alive after the 10 years of the follow-up (Figure 3A, B). On the contrary, only 58% of patients displaying MMP-2 positivity and oestrogen or progesterone receptor negativity were alive at that time. These differences were statistically highly significant, suggesting that these patient groups might need more attention in further studies. The patient group is small in percentages (about 4% of all breast carcinoma patients), but interesting both biologically and clinically. The number of those patients still exceeds the number of patients representing many more uncommon carcinoma types. The regulation of MMP-2 by female sex hormones may be an yet unknown mechanism which could explain this result. This conclusion indicates the need for further studies to explore the value of this enzyme in clinical decision-making.

In grade 2 and 3 tumours, MMP-2 correlated significantly with shortened RFS and overall survival (Table 2). It is interesting to note that, MMP-2 negativity in this patient group was a strong marker for a favourable prognosis. Barozzi *et al* (2002) reported that TGF-*α*, MMP-2 and IGF-II seem to be suitable candidates for a selective panel of markers designed to provide significant information with respect to the current pathologic staging system for patients with colorectal carcinoma.

In conclusion, we show here in a relatively large breast carcinoma patient group that MMP-2 immunoreactive protein is an independent prognostic indicator that might prove valuable in certain subgroups, such as patients with a receptor-negative breast carcinoma. The present data shows for the first time that MMP-2 negativity could serve as a marker for distinctly favourable prognosis in breast carcinoma patients. MMP-2 positivity is also shown to correlate to poor survival in node-negative breast carcinoma.
